# Understory Community Assembly Following Wildfire in Boreal Forests: Shift From Stochasticity to Competitive Exclusion and Environmental Filtering

**DOI:** 10.3389/fpls.2018.01854

**Published:** 2018-12-12

**Authors:** Bo Liu, Han Y. H. Chen, Jian Yang

**Affiliations:** ^1^CAS Key Laboratory of Forest Ecology and Management, Institute of Applied Ecology, Chinese Academy of Sciences, Shenyang, China; ^2^Faculty of Natural Resources Management, Lakehead University, Thunder Bay, ON, Canada; ^3^Key Laboratory for Humid Subtropical Eco-geographical Processes of the Ministry of Education, Fujian Normal University, Fuzhou, China; ^4^Department of Forestry and Natural Resources, University of Kentucky, Lexington, KY, United States

**Keywords:** chronosequence, community assembly, environmental filtering, functional diversity, trait conservatism, trait dispersion, phylogenetic diversity, overdispersion

## Abstract

Understory vegetation accounts for the majority of plant species diversity and serves as a driver of overstory succession and nutrient cycling in boreal forest ecosystems. However, investigations of the underlying assembly processes of understory vegetation associated with stand development following a wildfire disturbance are rare, particularly in Eurasian boreal forests. In this study, we measured the phylogenetic and functional diversity and trait dispersions of understory communities and tested how these patterns changed with stand age in the Great Xing'an Mountains of Northeastern China. Contrary to our expectation, we found that understory functional traits were phylogenetically convergent. We found that random patterns of phylogenetic, functional, and trait dispersions were dominant for most of our surveyed plots, indicating that stochastic processes may play a crucial role in the determination of understory community assembly. Yet, there was an evidence that understory community assembly was also determined by competitive exclusion and environmental filtering to a certain degree, which was demonstrated by the observed clustered phylogenetic and functional patterns in some plots. Our results showed that phylogenetic diversity significantly decreased, while functional diversity increased with stand age. The observed shift trends in phylogenetic and functional patterns between random to clustering along with stand age, which suggested that understory community assembly shifted from stochasticity to competitive exclusion and environmental filtering. Our study presented a difference to community assembly and species coexistence theories insisted solely on deterministic processes. These findings indicated that Eurasian boreal understory communities may be primarily regulated by stochastic processes, providing complementary evidence that stochastic processes are crucial in the determination of community assembly both in tropical and boreal forests.

## Introduction

Wildfires are common and widespread in ecosystems, serving as a global “herbivore” in the determination of plant distribution, and therefore, community composition (Bond and Keeley, 2005). Wildfire is a potent evolutionary force for fire-tolerant and fire-dependent plant species (Bond and Keeley, 2005). Functional traits that enable rapid colonization and efficient post-fire regeneration are crucial for the successful establishment and persistence of fire-adaptive plant communities (Pausas et al., 2004; Pausas and Keeley, 2014). Although global burned areas have declined over the last two decades due to anthropogenic activities (Andela et al., 2017), fire frequency and severity are anticipated to increase in many ecosystems as a consequence of projected climate change in the coming decades (Stephens et al., 2013; Jolly et al., 2015). Thus, an understanding of how forest communities are assembled following wildfire, is useful to land managers in the preparation of post-fire strategies for vegetation regeneration and future fuel management.

Two core processes have been proposed to explain vegetation assembly following stand-replacing disturbances (e.g., wildfire, wind, logging), namely stochastic processes and deterministic processes. The stochastic class emphasizes the importance of dispersal limitations and stochastic demographics in determining community assembly (Bell, 2000; Hubbell, 2001). In contrast, the deterministic class assumes that plant communities are generally driven by two opposing forces: environmental filtering, which gives rise to co-occurring species that are intimately related under similar environmental conditions, and competitive exclusion that decreases the similarity of co-occurring species (Weiher et al., 1998; Chesson, 2000; Cornwell et al., 2006). Previous studies have suggested that stochastic processes are dominant in the determination of community assembly in tropical forests (Condit et al., 2002; Chase, 2010), while niche based deterministic processes are predominant in mid-latitude temperate regions (Clark and McLachlan, 2003; Gilbert and Lechowicz, 2004).

**Figure 1:**
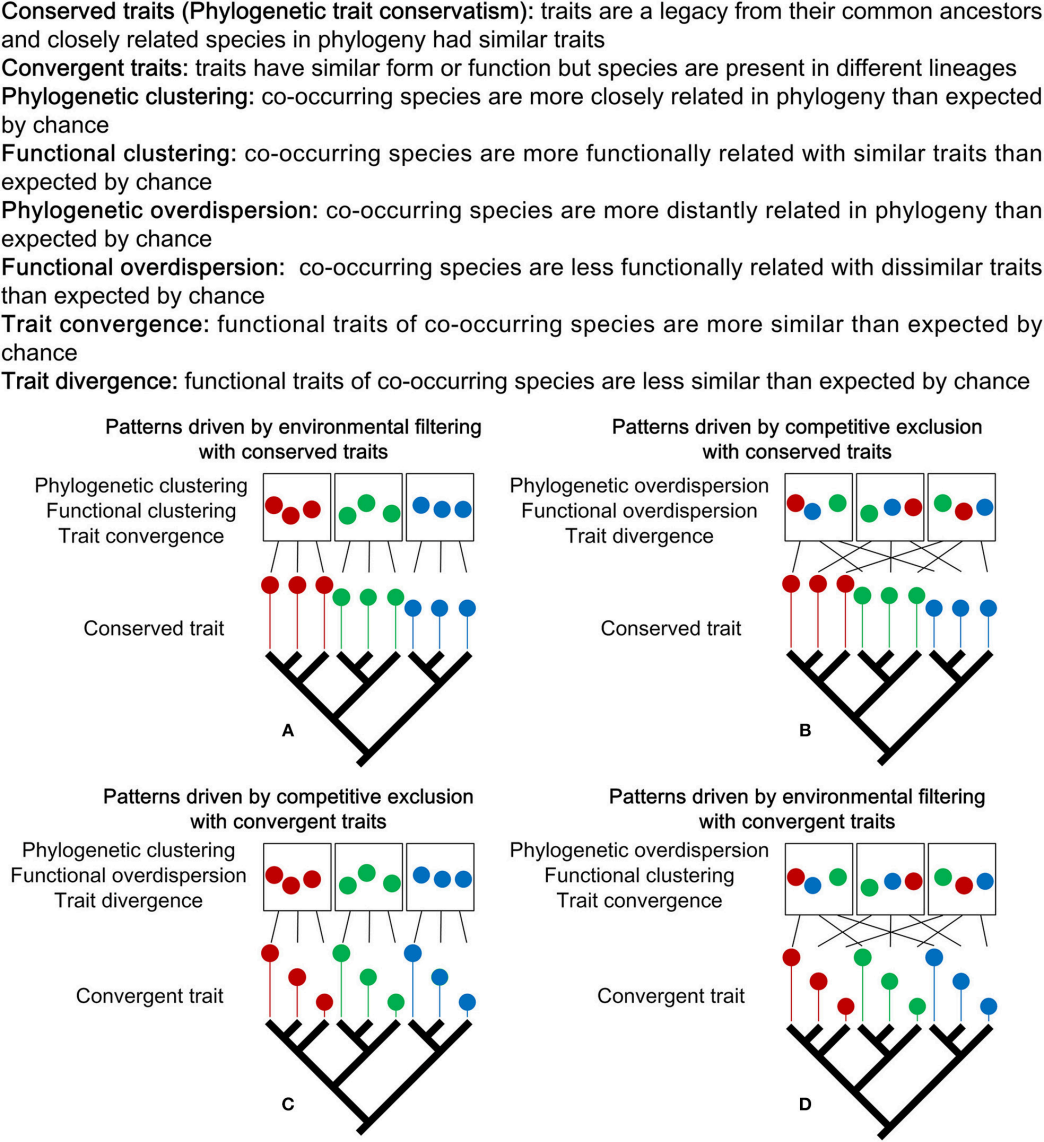
Environmental filtering and competitive exclusion give rise to opposite expectations about the phylogenetic, functional and trait patterns. Which ecological force can be invoked to explain a given phylogenetic, functional and trait patterns depends on whether trait evolution is conserved or convergent. When functional traits are phylogenetically conserved, environmental filtering drives clustering patterns **(A)** and competitive exclusion drives overdispersion patterns **(B)**. When functional traits are phylogenetically convergent, competitive exclusion drives phylogenetic clustering, functional overdispersion and trait divergence patterns **(C)**, and environmental filtering drives phylogenetic overdispersion, functional clustering and trait convergence patterns **(D)**. In each figure, there are three communities represented by rectangles. Within each community, colored circles represent species. Black lines represent the source of species from phylogenetic tree for each community. Colored lines with different length represent traits with different values. Figure adapted from Webb et al. (2002), Cavender-Bares et al. (2004), Kembel (2009), and Bernard-Verdier et al. (2012).

Over the last two decades, phylogenetic- and functional trait-based approaches were increasingly adopted for studies of tropical and temperate forest community assembly; however, there have been few for boreal forests (Kraft and Ackerly, 2010; Swenson et al., 2012a; Wang et al., 2015). Phylogenetic and functional patterns could reflect different ecological processes acting on community assembly (e.g., environmental filtering and competitive exclusion) with diversified evolution of functional traits (Figure 1). Studies have shown that determining the evolutionary patterns of functional traits is the prerequisite to interpreting the mechanisms of community phylogenetic and functional structure (Uriarte et al., 2010; Bennett et al., 2013; Fritschie et al., 2014). When functional traits are phylogenetically conserved (i.e., closely related species are ecologically similar with traits being a legacy from their ancestors), environmental filtering generally results in phylogenetic and functional clustering (Figure 1A); while competitive exclusion results in phylogenetic and functional overdispersion (Figure 1B) (Webb et al., 2002; Cavender-Bares et al., 2004; Kembel, 2009). In addition, environmental filtering will drive functional traits of co-occurring species to be more similar than expected by chance, namely trait convergence (Weiher et al., 1998; Cornwell et al., 2006; Grime, 2006) (Figure 1A). Conversely, competitive exclusion will drive functional traits of co-occurring species to be less similar than expected by chance, namely trait divergence (Figure 1B) (Chesson et al., 2004; Wilson, 2007; Wilson and Stubbs, 2012). When functional traits are phylogenetically convergent (i.e., species presented in different lineages have similar functional traits), environmental filtering would generate phylogenetic overdispersion, functional clustering and trait convergence (Figure 1C), while competitive exclusion will generate phylogenetic clustering, functional overdispersion and trait divergence (Figure 1D) (Cavender-Bares et al., 2004; Kembel, 2009; Uriarte et al., 2010; Bennett et al., 2013; Fritschie et al., 2014).

Previous studies have reported that stand age imparts potent influences on plant community assembly, due to the time required for colonization, and changes in resource availability as the stand develops (Hart and Chen, 2006; Verdú and Pausas, 2007; Kumar et al., 2017). According to the observed temporal changes in phylogenetic or functional patterns, successional studies have demonstrated that community assembly processes are altered as stands develop following stand-replacing disturbances (Norden et al., 2012; Purschke et al., 2013; Li et al., 2015; Muscarella et al., 2016). Stands at early successional stages are typically dominated by shade intolerant, nutrient demanding, and fast growing species, which results in phylogenetic and functional clustering, due to the availability of abundant resources subsequent to a disturbance (Verdú et al., 2009; Letcher et al., 2012). In contrast, late-successional communities are more often dominated by shade tolerant, slow growing and distantly related species with dissimilar functional traits, which are characterized by phylogenetic and functional over dispersion caused by competitive exclusion as resources become limited (Verdú et al., 2009; Letcher et al., 2012). Such transformation has been generally attributed to shifts in community assembly processes, from environmental filtering to competitive exclusion (Purschke et al., 2013).

To date, much research has primarily focused on overstory tree community assembly (Kooyman et al., 2011; Whitfeld et al., 2012), yet little empirical work exists for understory species (Azeria et al., 2011). In boreal region, forest understory communities constitutes the majority of plant diversity, serving as a critical driver for nutrient cycling and overstory succession (Nilsson and Wardle, 2005; Hart and Chen, 2006). Therefore, understanding the underlying mechanisms of understory community assembly may provide complementary information toward the elucidation of community assembly mechanisms following disturbances in boreal forest ecosystems. Here, we attempted to infer understory assembly processes from the temporal trends of phylogenetic and functional patterns coupling with trait dispersion in boreal forest. We surveyed understory species abundance and measured key functional traits over a chronosequence of 200 years following wildfire in a larch forest of Northeastern China. Specially, we tested phylogenetic trait conservatism to determine how understory functional traits evolved through quantifying phylogenetic signal and expected that understory functional traits were phylogenetically conserved. We tested the effects of stand age (years since fire) on understory phylogenetic and functional diversity and trait dispersion. We expected that phylogenetic and functional diversity and trait dispersion would change with stand age due to its strong control in the shifting of understory species composition (Hart and Chen, 2008; Kumar et al., 2017). By comparing the phylogenetic and functional patterns and trait dispersion of understory vegetation, we assessed the changes in relative importance of environmental filtering and competitive exclusion with stand development. We hypothesized that increasing stand age would promote phylogenetic and functional overdispersion and trait divergence as a result of increasing competitive exclusion (Spasojevic and Suding, 2012; Purschke et al., 2013).

## Materials and Methods

### Study Area

The present study was conducted at the southern margin of the Eurasian boreal forest, which is located in the Great Xing'an Mountains of Northeastern China. This study area is a mountainous region with elevations that range from 239 m in the Northeast, to 1,488 m in the Southwest. The climate in this region is typical terrestrial monsoon with a mean annual temperature of −4.4°C, ranging from −2.7°C to −5.3°C, and average annual precipitation ~500 mm. Fire comprises the primary natural disturbance, with a mean fire return interval of approximately 120–150 years (Chang et al., 2007). The typical vegetation type of this region belongs to the cool temperate coniferous forest (Zhou, 1991). The most dominant species is larch (*Larix gmelini* (Rupr.) Kuzen.), which is widely distributed in this region; typically forming pure stands. Birch (*Betula platyphylla* Suk.) is the widely distributed broadleaf species that intersperses in the larch forests at xeric sites. Pine (*Pinus sylvestris* L. var. *mongolica* Litv.), spruce (*Picea koraiensis* Nakai), aspen (*Populus davidiana* Dode, *Populus suaveolens* Fisch.) and willow (*Chosenia arbutifolia* (Pall.) A. Skv.) are also interspersed in larch forests, with a small area of distribution (< 2%). The most diverse component of the boreal forests is the understory vegetation, where common understory plants include *Betula fruticosa* Pall., *Rhododendron dauricum* L., *Vaccinium uliginosum* L., *Carex schmidtii*, and *Chamerion angustifolium*.

### Sampling Design

To examine the effects of stand age on understory vegetation, stands originating subsequent to wildfire were selected using a chronosequence approach. The chronosequence approach is recommended for the investigation of successional processes, over decadal to millennial time scales (Walker et al., 2010). Based on available fire-originating stands within the study area, we selected seven age classes of fire-originating stands, representing early stand initiation (4-year), late stand initiation (14-year), early stem exclusion (27-year), late stem exclusion (55-year), early canopy transition (76-year), late canopy transition (98-year), and gap dynamic (203-year) stages of stand development (Chen and Popadiouk, 2002). All stands were sampled on well-drained brown coniferous forest soil, which is the dominant soil type in this region (Gong, 2001). The selected stands were visually homogeneous in terms of stem density and composition within each stand age.

For the sampling of stands ≤ 50 years old, time since last fire was determined according to the fire occurrence records, which have been reported since 1965 (Liu et al., 2012). For the sampling of stands >50 years old, we confirmed fire to be the primary disturbance factor based on the black carbon in soil and the burned stump for forest stands over 50 years old. The stands we selected had cohorts of overstory seedlings and trees with similar age. Stand ages were determined through dendrochronology analysis (Chen et al., 2013). We selected larch to determine the time since fire for all sampled stands. For each stand, three to five trees were selected, and a core was extracted at breast height (1.3 m above root collar) from each tree. The cores collected in the field were stored and transported in plastic straws. In the laboratory, the cores were mounted on grooved, wooden core strips and sanded to make the growth rings visible. Subsequently, we counted the tree rings using a hand-held magnifier until the same number was obtained following three successive counts. In order to precisely represent stand age, we corrected the tree ages by adding 8 years to the ring counts made at breast height, accounting for the number of years required by trees to grow to breast height.

### Field Survey

A field survey was conducted during the peak vegetation cover from July to August. For each sampled stand, we randomly established a 400 m^2^ square plot for all measurements. In order to ensure accessibility and to avoid edge effects, each plot was selected within a walking distance of 50–2,000 m from a road. For stands of >4 years, the diameters at breast height (1.3 m above root collar) of all trees within the plot were measured and recorded. Tree and sapling density and basal area were summed to the plot level and calculated per hectare (Table S1). For stands in the 4-year age class, seedling basal areas were not reported here due to negligible values.

According to previous studies (Chipman and Johnson, 2002; Hart and Chen, 2008), the understory vegetation was surveyed in four randomly allocated 1 × 1 m quadrats. The percentage of cover for each shrub and herb species < 1.3 m in height within each quadrat was visually estimated (Mueller Dombois and Ellenberg, 1974). Specimens of any unidentified species were collected in the field and transferred to the Herbarium of Northeast China for identification. Species nomenclature followed the Flora Republicae Popularis Sinicae (http://frps.eflora.cn) and Flora of China databases (http://foc.eflora.cn/).

### Trait Selection and Measurements

Six morphological functional traits were selected to characterize the vegetative phase of understory vegetation according to Pérez-Harguindeguy et al. (2013). Leaf area (LA, mm^2^) is a major determinant of the ability of a species to sequester light resource, which can affect the photosynthetic rate. Leaf carbon content (LCC, %) and leaf dry matter content (LDMC, %) are related to physical plant resistance, leaf life span, and relative growth rate. Leaf nitrogen content (LNC, %) is a key foliar trait, which is strongly correlated with nutritional quality, the photosynthetic rate, and productivity. Plant height (PH, cm) is employed to quantify the light that is available for capture by understory plants. Specific leaf area (SLA, cm^2^/g) represents strategies for plant growth and survival, such as structural investment, leaf life spans, and photosynthetic rates. Functional trait values were determined following the new handbook for worldwide functional traits measurement (Pérez-Harguindeguy et al., 2013). We sampled at least five individuals for each observed species in each plot. The individuals we selected were reproductively mature and visually healthy. Following measurements, functional trait values were averaged at the species level for each sample plot as interspecific variability inclines to exceed intraspecific variability for understory traits (Burton et al., 2017). All understory species and the values of functional traits for each species were listed in the Table S2.

### Measurement of Trait Phylogenetic Conservatism

Before quantifying phylogenetic conservatisms for the measured functional traits, a phylogenetic tree for our study species was constructed using the online tool *Phylomatic* (http://phylodiversity.net/phylomatic/). This program generates a megatree with modern family and genus names based on previously published phylogenies (Phylogenetic tree version: zanne2014) (Zanne et al., 2014). Family and genus name resolutions are based on the Angiosperm Phylogeny Website (Stevens, 2001).

To assess the degree of species phylogenetic trait conservatism, we employed a widely used Blomberg's *K* statistic to quantify the phylogenetic signals of the six continuous traits (Blomberg et al., 2003). *K* was the ratio of the mean squared error of the tip data measured from the phylogenetically correct mean (*MSE*_0_), divided by the mean squared error of the data (*MSE*), which was quantified using the phylogenetic variance-covariance matrix, derived from the candidate tree. Afterward, *K* is calculated as:

(1) $$\begin{array}{r} K=observed\frac{{MSE}_{0}}{MSE}/expected\frac{{MSE}_{0}}{MSE} \end{array}$$

*K* is a continuous value, ranging from zero to infinity. When the *K* value is close to zero (p > 0.05), it implies a random or convergent pattern of trait evolution, and a weak phylogenetic signal. When the *K* value is close to one (*p* < 0.05), it implies that there is a strong phylogenetic signal, and that a trait has evolved based on the Brownian motion model. When the *K* value is higher than 1 (*p* < 0.05), it indicates a strong phylogenetic signal of traits, and closely related species are more similar than expected under a Brownian motion model of trait evolution. The *K* values were calculated in R 3.4.1 (R Development Core Team, 2017) using the “*picante*” package (Kembel et al., 2010). Additionally, we projected phylogenetic tree into trait space using traitgram and extended this to incorporate uncertainty about ancestral trait values along branches and at nodes, which was performed in R with package “*phylotools*” (Ackerly, 2009; Revell, 2013).

### Measurement of Phylogenetic and Functional Diversity and Trait Dispersion

The phylogenetic diversity within individual plots at each stand age was quantified via the mean pairwise distance (MPD), which measures the sum of the branch lengths of all co-occurring species within each community (Webb, 2000; Webb et al., 2002). We performed this analysis by using the phylogenetic dendrogram and weighted the pairwise distances among species by their relative coverage. An identical framework was utilized to quantity functional diversity using the trait dendrogram from Euclidean trait distances, referred to as mean pairwise functional distance (MFD) (Li et al., 2015). Community weighted trait variance (CWV) within individual plots at each stage was employed to depict trait dispersion (Bernard-Verdier et al., 2012), which was computed for each trait in each plot as follows:

(2) $$CWV=\overset{S}{\underset{i=1}{\sum^{\text{​}}}}p_{i}\times {\left(x_{i}\text{-}CWM\right)}^{2}$$

where CWV is the community weighted trait variance for a given functional trait, *p*_*i*_ is the relative abundance (percent coverage) of species *i* (*i* = 1, 2, …, S), *x*_*i*_ is the trait value of species *i*, and CWM is the community weighted mean trait value of species *i*. The community weighted mean trait value (CWM) for each functional trait (Garnier et al., 2004) was calculated as follows:

(3) $$\begin{array}{r} CWM=\overset{S}{\underset{i=1}{\sum }}p_{i}x_{i} \end{array}$$

where CWM is the community weighted mean value for a given functional trait, *p*_*i*_ is the relative abundance (percent coverage) of species *i* (*i* = 1, 2, …, S), and *x*_*i*_ is the trait value of species *i*. In order to account for the potential scale sensitivities of community patterns, all calculations of individual plots were replicated at a larger spatial scale by summing the plot composition within each stand age (*n* = 7).

### Null Model Testing

The null model was employed to determine whether the observed value of a metric varied from its random expectations. In order to achieve this goal, we implemented three null models. Null model 1 and 2 were generated through shuffling the names of taxa across the tips of the phylogenetic dendrogram and trait dendrogram, respectively (Kembel, 2009). Null model 3 was constructed to test the hypothesis that species abundance (percent coverage) within a community is randomly distributed with respect to trait values through shuffling the abundance values with unchanged traits (Mason et al., 2008; Bernard-Verdier et al., 2012). We then calculated the standardized effect size (SES) of MPD, MFD, and CWV as follows:

(4) $$\begin{array}{r} {SES}_{}=\frac{{Metric}_{observed}\text{-mean}\left({Metric}_{null}\right)}{sd\left({Metric}_{null}\right)} \end{array}$$

where *Metric*_*observed*_ is the observed value of MPD, MFD, and CWV, *Metric*_*null*_ is the mean values of random assemblages, and *sd(Metric*_*null*_*)* is the standard deviation of the random value. The negative values of SES.MPD and SES.MFD with low quantiles (*p* < 0.05) indicated smaller phylogenetic or functional distances among co-occurring species than expected by chance, namely, significant clustering. In contrast, positive values with high quantiles (*p* > 0.95) indicated greater phylogenetic or functional distances among species than expected by chance, namely, significant overdispersion (Webb et al., 2002). For SES.CWV, the negative values with low quantiles (*p* < 0.05) indicated trait convergence, whereas positive values with high quantiles (*p* > 0.95) indicated trait divergence. Non-significant positive or negative values indicated observed values close to the median of the random dispersion. The metrics of SES.MPD, SES.MFD, and SES.CWV were all calculated in R using the “*picante*” package with an “*abundance.weighted*” argument (Kembel et al., 2010).

### Statistical Analysis

The differences in SES.MPD, SES.MFD, and SES.CWV among stand ages were assessed using a non-parametric approach, as our data failed to meet the assumptions of normality and homogeneous variances. We employed rank-based one-way ANOVA to test the effects of stand age on SES.MPD, SES.MFD, and SES.CWV. These analyses were performed in R using the “*oneway.rfit*” function with “*Rfit*” package (Kloke and McKean, 2012). The significance of the differences among stand ages was tested by Dunn's *Post Hoc* test via the “*dunn.test*” function in R using the “*pgirmess*” package (Giraudoux, 2017). We used chi-squared tests to detect whether and how phylogenetic and functional patterns and trait dispersions changed from early successional stage (4- and 14-year age classes combined) to the late successional stage (98- and 203-year age classes combined).

## Results

### Trait Conservatism and Evolution

All measured traits exhibited small degrees of trait conservatism, with *K* values ranging from 0.083 for LDMC, to 0.218 for LCC (Table 1). This indicated that these traits were largely phylogenetically convergent, in contrast to the traits that were evolved under a Brownian motion model (all trait *K* values < 1; Table 1). Among these traits, four functional traits (LA, LCC, LNC, and SLA) were more conserved than the those predicted by a random association (four traits with *p* < 0.05; Table 1), whereas LDMC and PH were closer to zero, which corresponded to a random pattern of evolution. Mapping the six functional traits on the phylogenetic tree, we found functional traits displayed significant differences among phylogenetic related species (Figure S1). LA values of most species exhibited a widely range of 14.21–4,000 mm^2^, which was far away from the estimated ancestral value of LA (1,228 mm^2^) for all species (Figure S1a). As well, SLA had a widely range of values from 150 to 350 cm^2^/g, while the ancestral trait value is 234 cm^2^/g (Figure S1f). Conversely, LCC, LDMC, LNDC, and PH displayed a relatively narrow range of trait values, which were close to their estimated ancestral ancestral values (Figures S1b–e).

**Table 1:** Six measured species functional traits: leaf area (LA), leaf carbon concentration (LCC), leaf dry matter content (LDMC), leaf nitrogen concentration (LNC), plant height (PH), and specific leaf area (SLA).

**Trait**	**Type of variable**	**Mean (SE)**	***K***	***p***
LA	Continuous (mm^2^)	1921.06 (326.89)	0.176	0.022
LCC	Continuous (%)	43.20 (0.56)	0.218	0.001
LDMC	Continuous (%)	31.13 (0.97)	0.083	0.445
LNC	Continuous (%)	1.71 (0.08)	0.139	0.032
PH	Continuous (cm)	28.59 (3.10)	0.087	0.348
SLA	Continuous (cm^2^/g)	284.32 (16.78)	0.158	0.047

*The phylogenetic signal was measured using the K statistic. K values closer to 1 indicate a phylogenetic signal similar to a Brownian motion model of trait evolution. Values >1 indicate a strong phylogenetic signal, and values < 1 indicate a weak phylogenetic signal. P-values < 0.05 indicate that traits have a greater phylogenetic signal than the traits predicted by random association*.

### Phylogenetic and Functional Diversity and Trait Dispersion of Environmental Filtering and Competitive Exclusion

In the 76- and 203-year age classes, more than half of the plots had negative SES.MPD values, which were significantly different from those expected via the null model, indicating a clustered phylogenetic structure (Table 2). In other age classes, more than half, and even all plots, had SES.MPD values that were not significantly different from those expected by the null model, which indicated a randomly distributed phylogenetic structure (Table 2). The clustered patterns of phylogenetic structure were driven by competitive exclusion, as the traits were largely convergent. In the 4- and 55-year age classes, more than half of the plots had negative SES.MFD values, which were significantly different from those expected by the null model. This indicated a clustered functional structure that was driven by environmental filtering as the traits were largely convergent (Table 2). For other age classes, more than half, and even all plots, had SES.MFD values that were not significantly different from those expected by the null model, indicating a randomly distributed functional structure (Table 2). For the six measured functional traits, more than two-thirds, and even all plots had SES.CWV values that were not significantly different from those expected by the null model across all age classes, indicating random trait dispersions for most plots (Table S3).

**Table 2:** Percentage of clustering, random, and overdispersion patterns for phylogenetic (SES.MPD) and functional (SES.MFD) structures in each age class.

**Stand age**	**Phylogenetic structure**			**Functional structure**		
	**Clustering**	**Random**	**Overdispersion**	**Clustering**	**Random**	**Overdispersion**
4	0	100	0	56	44	0
14	0	100	0	33	67	0
27	25	75	0	0	100	0
55	50	50	0	67	33	0
76	83	17	0	8	92	0
98	42	58	0	33	67	0
203	58	42	0	25	75	0

### Phylogenetic and Functional Patterns and Trait Dispersions Across Stand Age

**Figure 2:**
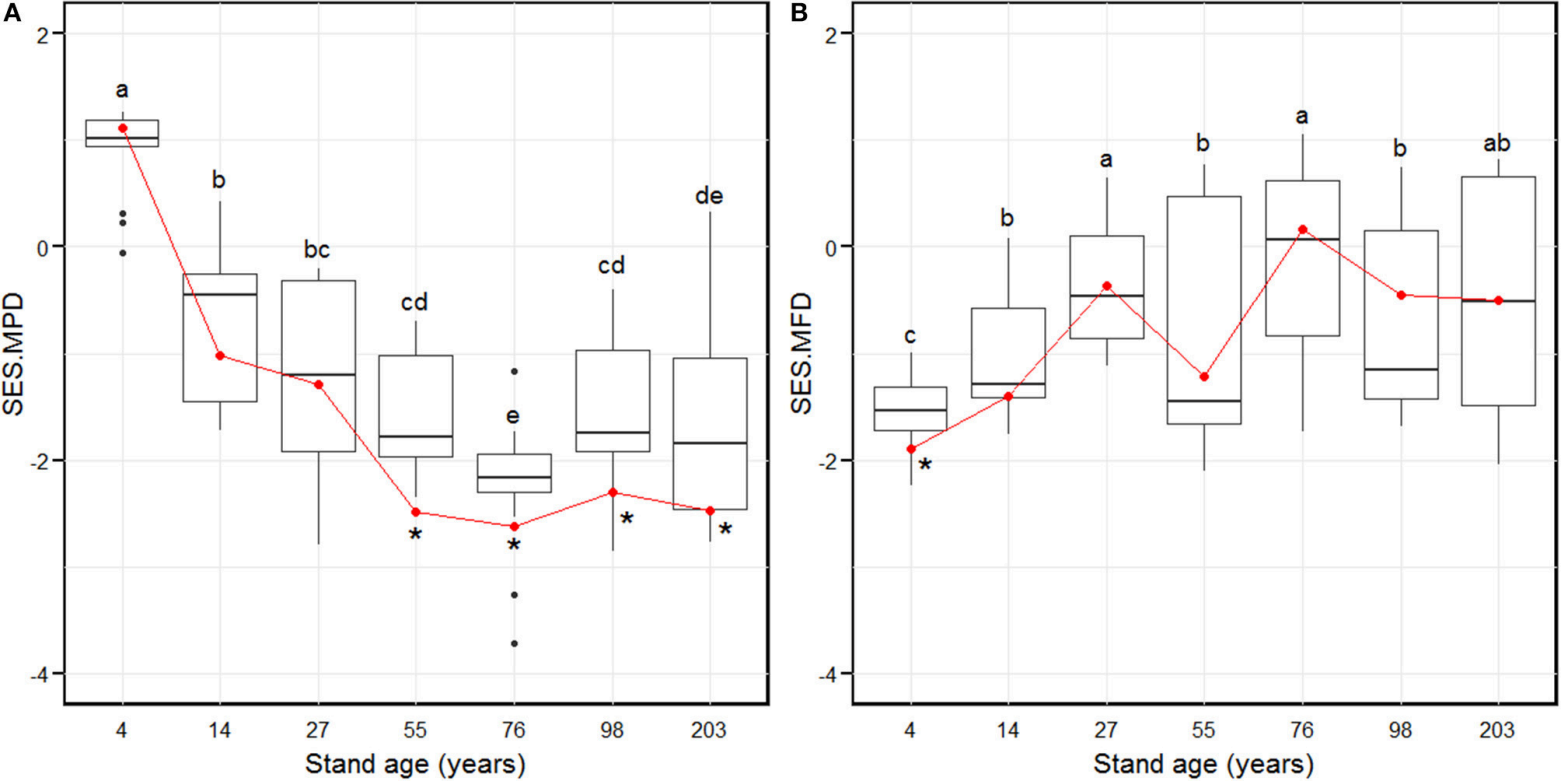
Standardized effect size of phylogenetic diversity (SES.MPD, **A**) and functional diversity (SES.MFD, **B**) along with stand age which were measured as the mean pairwise phylogenetic distance and the mean pairwise functional distance of the 88 plots over 203 years of succession, based separately on phylogeny and the functional dendrograms. Stand ages from 4 to 203 years represent early stand initiation, late stand initiation, early stem exclusion, late stem exclusion, early canopy transition, late canopy transition, and gap dynamics stages, respectively. The boxplot midlines correspond to the median value for each stand age; upper and lower hinges represent the first and third quartiles. Letters indicate significant differences of the mean SES values (*p* < 0.05) across the stand age. Red dots correspond to the SES values at a larger spatial scale by summing the plot composition for each stand age, and asterisks indicate a significant difference from the null model.

The SES.MPD decreased significantly from the 4-year age class and attained the lowest level in the 76-year age class (Figure 2A). Subsequently, the SES.MPD increased significantly and remained stable in the 98 and 203-year stages (Figure 2A). The SES.MFD attained the lowest value in the 4-year age class (Figure 2B). The SES.MFD then increased considerably and attained the highest level in the 27-year age class (Figure 2B). Further, the SES.MFD initially decreased and then increased between the 27 and 76-year age classes, with a further decrease and increase, from the 76 to 203-year age classes (Figure 2B). At the larger scale, by summing the plot composition for each age class, the SES.MPD was significantly different from the null model for the 55- to 203-year age classes (Figure 2A), and the SES.MFD was significantly different from the null model in only the 4-year age class (Figure 2B). The chi-squared test indicated that there was a pronounced shift of random phylogenetic pattern to clustering from early to late successional stages (Chi-squared = 18.2, *p* < 0.01), while there was no pronounced shift of functional pattern from early to late successional stage (Chi-squared = 1.6, *p* = 0.2).

**Figure 3:**
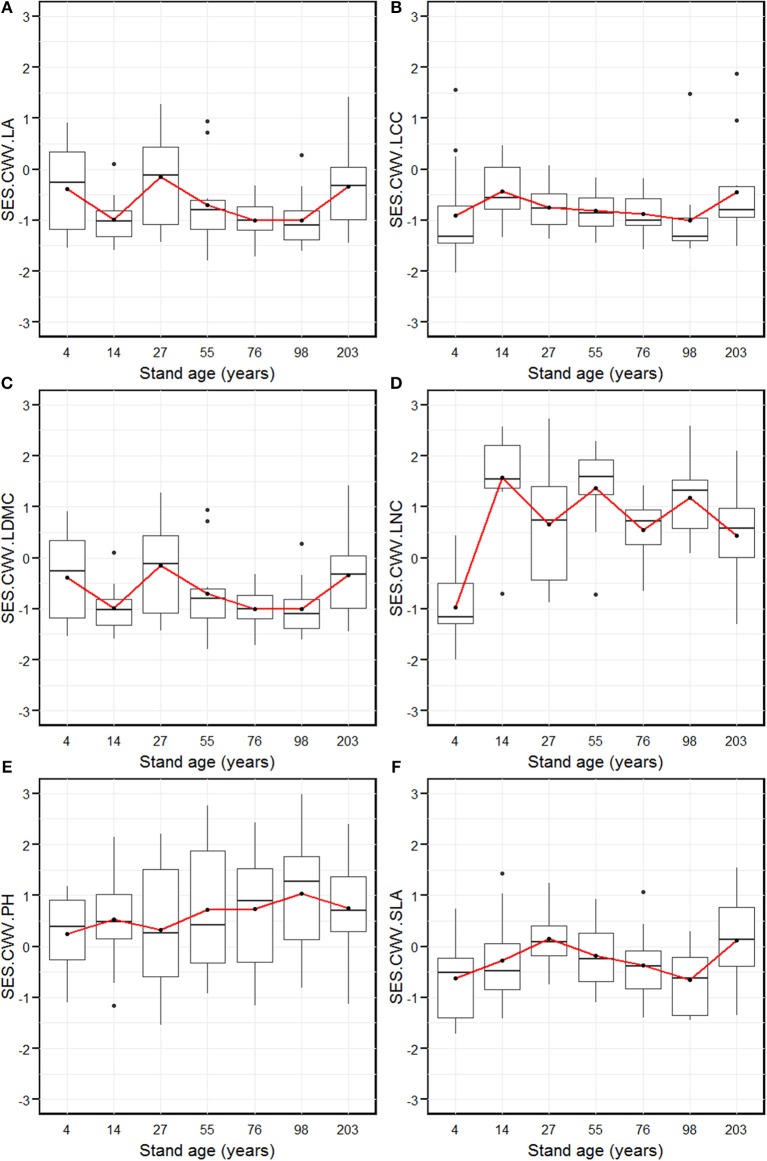
Standardized effect size of community weighted trait variance of leaf area (SES.CWV.LA, **A**), leaf carbon content (SES.CWV.LCC, **B**), leaf dry matter content (SES.CWV.LDMC, **C**), leaf nitrogen content (SES.CWV.LNC, **D**), plant height (SES.CWV.LA, **E**), and specific leaf area (SES.CWV.LA, **F**) along with stand age, which were measured by comparing observed CWV to a null model that was obtained by randomly shuffling the abundance values with unchanged traits. Stand ages from 4 years to 203 years represent early stand initiation, late stand initiation, early stem exclusion, late stem exclusion, early canopy transition, late canopy transition, and gap dynamics stages, respectively. The boxplot midlines correspond to the median value for each stand age; upper and lower hinges represent the first and third quartiles. Letters indicate significant differences of the mean SES values (*p* < 0.05) across the stand age. Red dots correspond to the SES values at the larger spatial scale by summing the plot composition for each stand age, and asterisks indicate a significant difference from the null model.

The SES.CWV revealed individualistic trends for the six measured functional traits along with stand age (Figure 3). The SES.CWV.LA (leaf area) decreased significantly from the 4-year age class, and attained the lowest level in the 14-year age class, which was followed by an increase to attain the highest level in the 27-year age class (Figure 3A). The SES.CWV.LA then decreased and attained the lowest level in the 76- and 98-year age classes, followed by an increase to attain the highest level in the 203-year age class. The SES.CWV.LCC (leaf carbon content) increased from the 4-year age class and attained the highest level in the 14-year age class, which then decreased and remained stable from the 55-year age class to the 98-year age class, followed by an increase to attain the highest level in the 203-year age class (Figure 3B). The SES.CWV.LDMC (leaf dry matter content) attained the highest value in the 4-year age class, which then decreased to attain the lowest level in the 76-year age class, followed by an increase to attain the highest level in the 203-year age class (Figure 3C). The SES.CWV.LNC (leaf nitrogen content) had the lowest value in the 4-year age class, which then increased and attained lowest level in the 27-year age class, followed by a regular increase and decrease from the 27-year age class to the 203-year age class (Figure 3D). The SES.CWV.SLA increased from the 4-year age class and attained the highest level in the 27-year age class, which then decreased and reached lowest level in the 98-year age class, followed by an increase to attain the highest level in the 203-year age class (Figure 3F). At the larger scale by summing the plot composition for each stand age, the values of SES.CWV.LA were significantly different from the null model, except in the 4- and 27-year age classes (Figure 3A). Similarly, the values of SES.CWV.LCC were significantly different from the null model in only the 203-year age class (Figure 3B), and the values of SES.CWV.LDMC were significantly different from the null model in the 4- and 14-year age classes (Figure 3C). The SES values of LNC, PH, and SLA were not significantly different from the null model across all age classes (Figures 3D–F).

## Discussion

In this study, we firstly tested the degree of phylogenetic trait conservatism for boreal forest understory through quantifying phylogenetic signal (Losos, 2008; Revell et al., 2008; Ackerly, 2009). According to previous studies, phylogenetically conserved traits may result in low rates of trait evolution; conversely, less phylogenetically conserved traits may result in a random or convergent pattern of trait evolution (Webb et al., 2002; Ackerly, 2004). The degree of phylogenetic trait conservatism has been widely tested in tropical and temperate forests, and studies in support of phylogenetic trait conservatism appear to be dominant (Ackerly, 2003). However, there were also counterexamples which suggested that key functional traits, especially leaf morphological traits, were evolutionarily labile for tree species (Kraft and Ackerly, 2010; Swenson et al., 2012b). In our study, the phylogenetic signals of four measured functional traits were >0 (*p* < 0.05) but < 1, which indicated that the key functional traits related to understory light capture strategies were less phylogenetically conserved (Ackerly, 2009). Furthermore, the traitgrams of the six functional traits implied that these functional traits were convergently evolved (Ackerly, 2009).

Contrary to our expectations, the phylogenetic and functional diversity of most plots were not significantly different from those expected by the null models, which indicated that most understory communities were randomly assembled (Webb et al., 2002; Kembel, 2009). These random patterns of co-occurring understory species might be a consequence of stochastic processes, such as external spatially random colonization and germination from the soil seed bank (Hubbell, 2001; Ulrich et al., 2016). Aside from random patterns, there was a clustered phylogenetic pattern, which might be caused by competitive exclusion as trait evolution was largely convergent (Cavender-Bares et al., 2004; Pausas and Verd,ú, 2010). The phylogenetic structure was inclined to shift from random to clustering, which suggested that the importance of competitive exclusion increased from early to late successional stages. In addition, we also found that the clustered functional pattern, which might be caused by environmental filtering as trait evolution was largely convergent (Cavender-Bares et al., 2004). Although we observed clustered functional patterns in the 4- and 55-year age classes, we failed to observe shifts between clustered and random patterns, from early to late successional stages. This suggested that environmental filtering had only a minimal impact on understory assemblages in some specific age classes. We found that the community weighted trait variance for most communities were equal to those expected by the null model, which indicated that random trait dispersion was dominant in random community assemblages (Bernard-Verdier et al., 2012).

Our study suggested that wildfire substantially altered understory phylogenetic and functional diversity, and community trait dispersions. In our study, phylogenetic diversity decreased while functional diversity increased along with stand age, which showed different temporal trends compared with previous studies. For instance, Li et al. (2015) showed a unimodal relationship of phylogenetic diversity, and a linear relationship of functional diversity, along with stand age, respectively. In contrast, Purschke et al. (2013) found that both the phylogenetic and functional diversity of grassland communities increased along with long-term succession. In addition, we found that phylogenetic and functional diversity exhibited different temporal trends compared with species richness (Figure S2), which initially increased and then decreased along with stand age (Hart and Chen, 2006). This suggested that phylogenetic and functional diversity had weak correlations with species richness in Eurasian boreal forests.

Our results failed to support the assumption that increased local phylogenetic diversity corresponded to increased local functional diversity. This might have been caused by the degree of phylogenetic trait conservatism of the measured functional traits in our study (Gerhold et al., 2015). In addition, we found that the community weighted trait variance for the five traits that we measured, changed significantly but with various temporal trends along with stand age. The differences in phylogenetic and functional diversity, and community weighted trait variance among stand age classes might be attributed to random species colorization and extinction (Lapiedra et al., 2015).

## Conclusion

Using both phylogenetic- and functional-centric approaches, we presented that the assembly and maintenance of understory communities were the sum of different ecological processes. Our study provided evidence that stochastic processes dominated in the control of boreal forest understory community assembly as shown by the observed random phylogenetic, functional and trait patterns for most plots. We also found that environmental filtering and competitive exclusion affected understory community assembly to a certain degree, which was indicated by the observed clustered phylogenetic and functional patterns for some plots. Our results demonstrated that understory community assembly following wildfire in boreal forests shifted from stochasticity to competitive exclusion and environmental filtering. Our study presented a difference to community assembly and species coexistence theories insisted solely on deterministic processes. We anticipated that our findings might provide complementary information toward the further elucidation of community assembly mechanisms following disturbances in boreal forest ecosystems.

## Author Contributions

BL, HC, and JY conceived this research. BL collected and analyzed data. BL, HC, and JY wrote the manuscript.

### Conflict of Interest Statement

The authors declare that the research was conducted in the absence of any commercial or financial relationships that could be construed as a potential conflict of interest.
