# Supplementary material for: Understory Community Assembly Following Wildfire in Boreal Forests: Shift From Stochasticity to Competitive Exclusion and Environmental Filtering
Source: Front Plant Sci. 2018 Dec 12;9:1854. doi: 10.3389/fpls.2018.01854 (PMC6315132; doi:10.3389/fpls.2018.01854)
Supplement: Supplementary file 1 [file Table_1.docx]

# SUPPLEMENTARY MATERIAL FOR ONLINE PUBLICATION ONLY

## **TABLE S1.** Overstory stand characteristics of 88 stands that were sampled in the boreal forests of the Great Xing’an Mountains. Values are means with SE in parentheses, and denote for larch and birch trees together. For the 4- and 203-year age classes, seedling basal area was not reported here due to small values.

| Age class  (year) | *n* | Tree density | Tree basal  area (m^2^/h^2^) | Seedling and sapling density | Seedling and sapling basal  area (m^2^/h^2^) |
| --- | --- | --- | --- | --- | --- |
| 4 | 16 | 0 | 0 | 5146(1761) | 0 |
| 14 | 12 | 0 | 0 | 4889 (1859) | 3.70 (2.04) |
| 27 | 12 | 2976 (343) | 15.56 (1.26) | 2202 (546) | 2.05 (0.47) |
| 55 | 12 | 2253 (163) | 16.42 (1.17) | 1477 (313) | 0.87 (0.14) |
| 76 | 12 | 3065 (147) | 26.61 (1.47) | 575 (179) | 0.54 (0.20) |
| 98 | 12 | 1743 (117) | 19.03 (1.16) | 1041 (526) | 0.71 (0.16) |
| 203 | 12 | 1272 (159) | 31.94 (3.65) | 0 | 0 |

## **TABLE S2.** Understory species observed in the field and the values of functional traits for each species. LA, LCC, LDMC, LNC, PH, and SLA denote for leaf area (mm^2^), leaf carbon content (%), leaf dry matter content (%), leaf nitrogen content (%), plant height (cm), and specific leaf area (cm^2^/g), respectively.

| Understory species | LA | LCC | LDMC | LNC | PH | SLA |
| --- | --- | --- | --- | --- | --- | --- |
| *Aquilegia oxysepala* | 1662.12 | 39.70 | 26.96 | 0.98 | 17.50 | 257.17 |
| *Artemisia tanacetifolia* | 3650.05 | 41.90 | 22.79 | 1.36 | 12.04 | 288.05 |
| *Betula rotundifolia* | 484.06 | 45.37 | 36.83 | 1.82 | 76.32 | 185.71 |
| *Caltha palustris* | 1267.58 | 40.02 | 21.00 | 1.24 | 8.00 | 298.25 |
| *Carex schmidtii* | 421.87 | 44.88 | 39.38 | 1.73 | 27.48 | 194.37 |
| *Chamerion angustifolium* | 1310.73 | 44.78 | 32.17 | 1.79 | 59.93 | 155.28 |
| *Convallaria majalis* | 1540.30 | 42.37 | 31.20 | 0.21 | 20.00 | 350.61 |
| *Deyeuxia angustifolia* | 1291.48 | 46.53 | 38.66 | 1.87 | 46.45 | 302.77 |
| *Fragaria orientalis* | 2796.26 | 43.73 | 34.27 | 1.40 | 12.06 | 353.20 |
| *Galium boreale* | 149.02 | 42.56 | 37.00 | 3.03 | 30.00 | 407.82 |
| *Geranium maximowiczii* | 1572.36 | 37.03 | 34.21 | 1.52 | 15.00 | 459.62 |
| *Iris uniflora* | 1663.66 | 41.31 | 24.00 | 2.09 | 24.94 | 305.78 |
| *Juniperus rigida* | 16.52 | 48.97 | 30.13 | 1.24 | 30.00 | 50.27 |
| *Lathyrus humilis* | 2855.56 | 42.34 | 30.02 | 2.44 | 24.39 | 293.18 |
| *Ledum palustre* | 81.50 | 49.42 | 41.57 | 2.09 | 30.54 | 111.69 |
| *Linnaea borealis* | 75.11 | 42.54 | 31.96 | 1.33 | 4.25 | 398.64 |
| *Maianthemum bifolium* | 1507.22 | 40.79 | 22.53 | 1.31 | 16.41 | 341.30 |
| *Ostericum maximowiczii* | 3069.84 | 43.28 | 24.94 | 1.61 | 27.10 | 258.61 |
| *Paris verticillata* | 960.45 | 41.03 | 33.00 | 1.73 | 20.00 | 300.14 |
| *Polygonatum odoratum* | 1315.56 | 43.24 | 22.00 | 1.52 | 15.00 | 386.93 |
| *Potentilla fruticosa* | 314.43 | 44.03 | 32.72 | 1.77 | 43.75 | 238.79 |
| *Pyrola incarnata* | 1014.88 | 46.45 | 35.35 | 1.17 | 6.29 | 171.47 |
| *Rhododendron dauricum* | 464.19 | 47.15 | 31.22 | 2.08 | 92.23 | 241.11 |
| *Rhododendron lapponicum* | 131.70 | 49.74 | 26.06 | 2.16 | 6.00 | 180.28 |
| *Ribes pauciflorum* | 3722.23 | 44.04 | 33.75 | 1.83 | 46.00 | 240.43 |
| *Ribes procumbens* | 1852.44 | 44.59 | 31.00 | 1.38 | 20.00 | 187.33 |
| *Rosa davurica* | 2714.50 | 42.10 | 39.32 | 1.64 | 26.73 | 240.79 |
| *Rubus arcticus* | 2655.74 | 41.97 | 30.02 | 1.71 | 11.00 | 396.29 |
| *Rubus sachalinensis* | 3694.58 | 43.21 | 37.81 | 1.64 | 31.66 | 212.80 |
| *Salix pentandra* | 439.10 | 45.79 | 32.20 | 2.38 | 10.67 | 277.33 |
| *Salix raddeana* | 681.78 | 42.41 | 35.18 | 2.16 | 56.94 | 215.72 |
| *Sanguisorba officinalis* | 4016.59 | 31.07 | 28.90 | 1.55 | 21.65 | 323.50 |
| *Saussurea sinuata* | 8622.81 | 40.48 | 35.00 | 1.77 | 32.50 | 574.85 |
| *Saussurea umbrosa* | 8117.70 | 37.78 | 14.21 | 1.19 | 19.99 | 345.15 |
| *Sorbaria sorbifolia* | 6891.35 | 43.79 | 30.52 | 1.59 | 64.85 | 285.38 |
| *Spiraea media* | 441.65 | 46.34 | 36.70 | 1.36 | 52.54 | 272.36 |
| *Vaccinium uliginosum* | 204.64 | 47.01 | 39.89 | 2.22 | 28.02 | 191.32 |
| *Vaccinium vitis-idaea* | 193.19 | 48.41 | 37.06 | 1.43 | 18.46 | 98.97 |
| *Vicia cracca* | 154.11 | 42.05 | 28.57 | 2.55 | 30.00 | 369.21 |
| *Vicia pseudorobus* | 3836.44 | 42.56 | 23.50 | 3.03 | 30.00 | 415.99 |
| *Viola acuminata* | 908.23 | 38.66 | 22.73 | 1.41 | 5.67 | 478.59 |

## **TABLE S3.** The percentage of standardized effect size of community weighted trait variance (SES.CWV) in each stand age. LA, LCC, LDMC, LNC, PH, and SLA denote for leaf area, leaf carbon content, leaf dry matter content, leaf nitrogen content, plant height, and specific leaf area, respectively.

| Stand age |  | 4 | 14 | 27 | 55 | 76 | 98 | 203 |
| --- | --- | --- | --- | --- | --- | --- | --- | --- |
| SES.CWV.LA | Convergent | 20 | 17 | 8 | 18 | 25 | 9 | 17 |
|  | Random | 80 | 83 | 92 | 82 | 75 | 91 | 83 |
|  | Divergent | 0 | 0 | 0 | 0 | 0 | 0 | 0 |
| SES.CWV.LCC | Convergent | 27 | 0 | 8 | 18 | 17 | 18 | 8 |
|  | Random | 66 | 100 | 92 | 82 | 83 | 82 | 92 |
|  | Divergent | 7 | 0 | 0 | 0 | 0 | 0 | 0 |
| SES.CWV.LDMC | Convergent | 0 | 0 | 0 | 0 | 0 | 0 | 8 |
|  | Random | 100 | 82 | 100 | 100 | 100 | 100 | 67 |
|  | Divergent | 0 | 8 | 0 | 0 | 0 | 0 | 25 |
| SES.CWV.LNC | Convergent | 20 | 0 | 0 | 0 | 0 | 0 | 0 |
|  | Random | 80 | 67 | 92 | 73 | 100 | 73 | 92 |
|  | Divergent | 0 | 33 | 8 | 27 | 0 | 27 | 8 |
| SES.CWV.PH | Convergent | 0 | 0 | 8 | 0 | 0 | 0 | 0 |
|  | Random | 100 | 92 | 84 | 91 | 92 | 91 | 75 |
|  | Divergent | 0 | 8 | 8 | 9 | 8 | 9 | 25 |
| SES.CWV.SLA | Convergent | 20 | 0 | 0 | 0 | 8 | 0 | 0 |
|  | Random | 80 | 100 | 100 | 100 | 92 | 100 | 92 |
|  | Divergent | 0 | 0 | 0 | 0 | 0 | 0 | 8 |

**
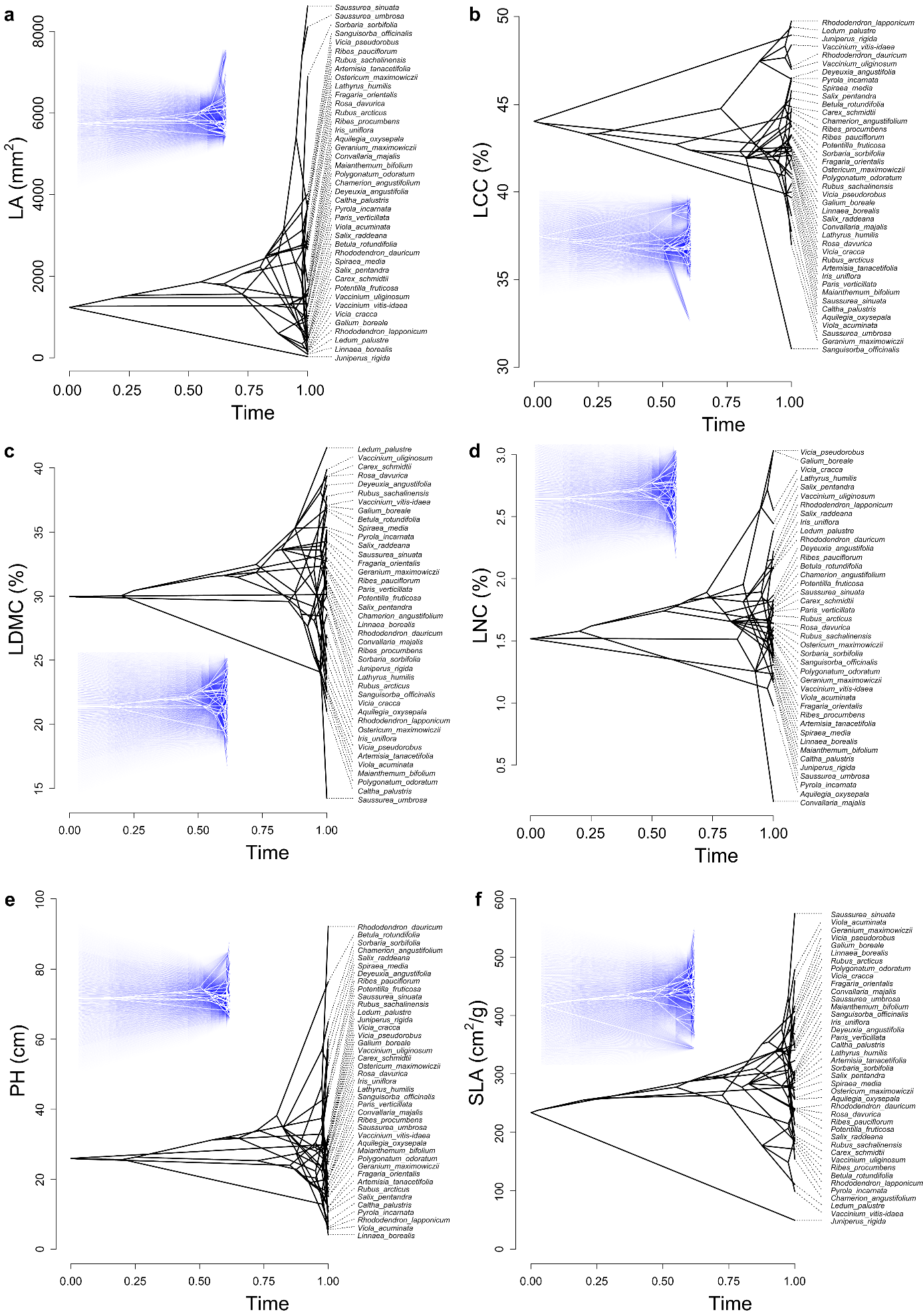
**

## **FIGURE S1.** Tratgram displayed the projections of the understory phylogeny into the space defined by functional traits of LA (a), LCC (b), LDMC (c), LNC (d), PH (e) and SLA (f) on y-axis and time since divergence from the root on x-axis. The vertical position of nodes and branches are computed via ancestral character estimation using maximum likelihood. The embedded images indicated uncertainty via increasing transparency of the plotted blue lines around the point estimates with the entire range showing the 95% confidence interval.


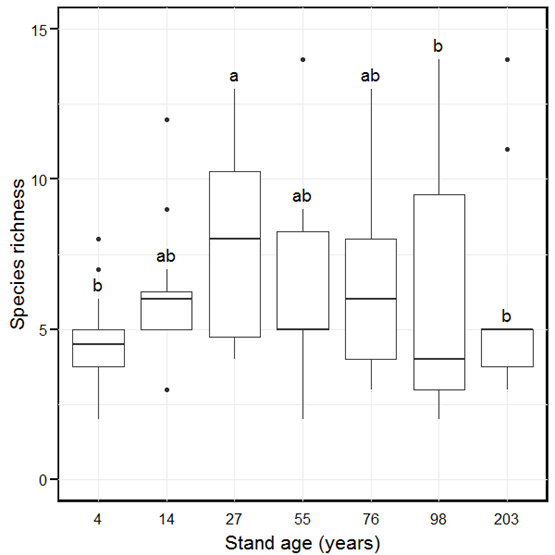


# **FIGURE S2.** The boxplot of species richness along with stand age. Letters indicate significant differences of the mean species richness (p < 0.05) across the stand age.
